# The minipig as a programmable translational platform for experimental oncofertility

**DOI:** 10.3389/fonc.2026.1981709

**Published:** 2026-10-15

**Authors:** Rodrigo Albertho Almeida Wedy, Lucas Damé Simões, Bruna Silveira Pacheco, Priscila Marques Moura de Leon, Fabiana Kömmling Seixas, Ligiane Leme, Luciano Abreu Brito, Ernesto Goulart, Maria Lucia Zaidan Dagli, Tiago Veiras Collares

**Affiliations:** 1 Cancer Biotechnology Laboratory, Center for Technological Development, Federal University of Pelotas, Pelotas, RS, Brazil; 2 INCT Bio^2^: Translational Biodiscovery and Biomodels/CNPq, Fortaleza, Brazil; 3 Human Genome and Stem Cell Research Center, Department of Genetics and Evolutionary Biology, Institute of Biosciences, University of São Paulo, São Paulo, SP, Brazil; 4 Department of Pathology, School of Veterinary Medicine and Animal Science, University of São Paulo, São Paulo, SP, Brazil

**Keywords:** fertility preservation, genome engineering, genomic provenance, minipig, oncofertility, oncopig, precision oncology, reproductive cancer

## Abstract

Experimental oncofertility remains fragmented across models of cancer biology, treatment-associated gonadotoxicity, fertility preservation, and reproductive recovery. We propose extending the Oncopig concept into a translational platform that reconnects these states within one genetically defined, cancer-bearing host. The Oncopig provides the oncologic backbone, combining programmable cancer in an immunocompetent large-animal host with clinically scaled intervention and longitudinal sampling. High-resolution porcine genomics, CRISPR-based genome engineering, somatic-cell nuclear transfer for line generation, comparative experimental pathology, and mature reproductive biotechnology extend this architecture toward reproductive endpoints. Here, programmability refers to deliberate specification of the cancer state—genotype, cell lineage or induction strategy, and timing—whereas the reproductive phenotype is measured longitudinally rather than programmed. Distant-cancer modules can test whether cancer status modifies gonadotoxicity, while reproductive-organ modules require disease-specific alleles, appropriate cell-of-origin targeting, and pathology-led validation. Because porcine and human reproduction align in several endocrine and gonadal processes but differ in cycle organization, implantation, placentation, and pregnancy, translation should emphasize process-level endpoints rather than numerical equivalence. The platform is particularly suited to unresolved questions involving targeted therapies and immunotherapies, preservation timing, and pediatric or prepubertal patients. Porcine reproductive biotechnology provides functional endpoints from gonadal tissue and gametes to embryos and offspring. Rather than replacing rodent or human-based models, the Oncopig framework adds an integrated translational layer for questions requiring clinically scaled intervention, longitudinal physiology, and functional reproductive recovery.

## 1 Experimental oncofertility needs biological continuity

Oncofertility is clinically organized around a continuous trajectory: cancer diagnosis, fertility counseling, preservation when indicated, treatment, survivorship, and reproductive follow-up (1, 2). In contrast, experimental oncofertility is typically fragmented across separate model systems. Tumor biology is usually investigated in cancer models, gonadotoxicity in healthy animals or isolated gonads, cryopreservation in reproductive systems, and recovery in separate transplantation or clinical cohorts. The patient experiences one biological history, whereas the laboratory commonly reconstructs that history from disconnected models.

This discontinuity creates a causal blind spot. Reproductive tissue collected before therapy may already be conditioned by the cancer-bearing host; treatment may alter not only germ-cell number but stromal, vascular, endocrine, immune, or genomic states; and preservation procedures can add another biological stress. Consequently, a post-treatment reproductive phenotype may reflect the interaction of cancer, host biology, therapy, preservation, and time rather than the independent effect of any one component. Evidence that some cancers are associated with altered pretreatment ovarian reserve or sperm molecular states, together with a recent mouse allograft study showing that non-reproductive tumors can impair ovarian function through systemic CXCL10-IL18R1 signaling, makes this distinction biologically plausible rather than semantic (3–5). That study establishes mechanistic small-animal evidence rather than large-animal validation. The Oncopig framework would extend this mechanistic evidence into a clinically scaled, immunocompetent cancer-bearing host in which tumor-conditioned reproductive injury can be linked to pharmacokinetics, serial endocrine and tissue sampling, fertility-preservation procedures, and later gamete or reproductive competence.

We therefore distinguish reproductive toxicology from experimental oncofertility. The former asks what an exposure does to a healthy reproductive system. The latter asks how reproduction changes across cancer, treatment, preservation, and recovery within the same disease-bearing host. The relevant experimental unit is the longitudinal cancer–host–reproductive system. We argue that a genetically defined minipig can make this unit experimentally accessible at a scale compatible with serial sampling, imaging, pharmacokinetics, surgery, ovarian and testicular procedures, and long-term reproductive assessment (6).

Biological continuity is particularly consequential in pediatric, adolescent, and young-adult oncology. Prepubertal patients may have no mature gametes available for banking, making ovarian- or testicular-tissue preservation the only tissue-based option that can be offered before gonadotoxic therapy; in adolescents and young adults, treatment urgency, changing reproductive potential, and survivorship can create a second decision point after therapy. These settings expose a limitation of endpoint-based models: tissue collected before treatment, tissue exposed *in vivo* to therapy, and tissue evaluated years later in a survivor are usually not linked experimentally. A longitudinal minipig model could preserve that linkage and test whether age, pubertal state, cancer status, and treatment history alter the biological quality of tissue intended for future fertility restoration (1, 7, 8).

## 2 Why the minipig, and why now?

The case for the minipig has changed. Its relevance now derives not only from anatomical and physiological similarity, but from the convergence of comparative genomics, next-generation sequencing, genome engineering, somatic-cell nuclear transfer, programmable cancer, and reproductive biotechnology. The 2012 pig genome created a comparative genomic framework for human disease; Sscrofa11.1 improved contiguity and annotation; PigGTEx added tissue-resolved regulatory information; and telomere-to-telomere assemblies now expose structural and repetitive regions that were previously inaccessible (9–12). Importantly, T2T-pig1.0 was assembled from a male Wuzhishan pig (Sus scrofa; NCBI BioProject PRJNA1139427), whereas foundational Oncopig development used a Minnesota miniature-swine background. It therefore demonstrates technical maturity rather than substituting for a model-specific reference genome. A dedicated platform would benefit from *de novo*, haplotype-resolved characterization of the actual minipig lineage being studied.

Reproductive translation also requires explicit species boundaries. Women and pigs are both continuously cycling spontaneous ovulators with follicular and luteal phases regulated by the hypothalamic-pituitary-ovarian axis, and their hormonal cycle organization is broadly comparable. Important differences remain: the porcine estrous cycle is approximately 18–23 days rather than a menstrual cycle, pigs do not menstruate, they are polytocous and typically ovulate multiple follicles, and uterine anatomy and luteolytic regulation differ (13, 14). Pregnancy endpoints require an additional distinction because pigs have diffuse epitheliochorial placentation, whereas humans have discoid hemochorial placentation (15). Male reproductive biology also supports process-level comparison but not temporal equivalence: the porcine seminiferous epithelial cycle is approximately 8.6-9.0 days versus about 16 days in humans, and sperm-production efficiency differs substantially between species (16). Accordingly, the strongest cross-species readouts are process-level measures such as gonadal histopathology, endocrine trajectories, follicular or spermatogenic injury and recovery, and gamete competence. Absolute timing, endocrine thresholds, implantation, placental transfer, pregnancy, and offspring outcomes should be interpreted as species-specific unless concordance is independently established.

The Oncopig established the complementary principle that cancer can be programmed in an immunocompetent pig through Cre-inducible KRAS^G12D^ and TP53^R167H^ alterations (17, 18). In this Perspective, the Oncopig is treated not as a fixed two-allele model for every cancer type, but as an established oncologic backbone: an immunocompetent, genetically controlled large-animal system on which disease-specific cancer modules can be built. Here, “programmable” refers specifically to deliberate control of the cancer state—genotype, cell lineage or induction strategy, and timing—not to the reproductive phenotype, which is observed longitudinally. Its relevance to oncofertility is now supported by a broader translational record: Oncopig tumors have been developed in multiple organs and interrogated with human-scale CT, MRI, ultrasound, fluoroscopy, serial biopsy, ablation, arterial drug delivery, and precision-treatment strategies (19–21). These capabilities matter because treatment modality, dose, route, combination, and timing can be tested in a tumor-bearing host rather than inferred from healthy-animal toxicology.

Genome engineering provides causal control. SCNT permits clonally selected, molecularly characterized donor cells to generate defined animals, whereas CRISPR enables direct, multiplex, and pathway-level editing (22–29).

SCNT should, however, be treated as a model-generation tool rather than the preferred generation for definitive reproductive phenotyping. Porcine SCNT is associated with incomplete epigenetic reprogramming and placental abnormalities in a subset of conceptions, creating a potential confound for reproductive endpoints (30). The preferred design is therefore to establish and molecularly validate the engineered line in SCNT founders, transmit the allele through conventional breeding, and perform primary reproductive phenotyping in genotype-defined F1 offspring; F2 animals can be used when allele segregation, lineage stabilization, or independent confirmation is required. Evidence that a cloned minipig can generate fertile F1 and F2 offspring supports the feasibility of this strategy but does not eliminate the need to separate founder-generation effects from disease- or therapy-associated reproductive phenotypes (31). This generation-specific precaution concerns SCNT-derived reproductive phenotyping and does not limit the Oncopig cancer platform itself.

Xenotransplantation has demonstrated the maturity of the porcine engineering toolkit: PERV inactivation, extensive germline engineering, and humanized donor pigs show that porcine genomes can be redesigned and functionally validated at whole-animal scale (26–29). Oncofertility does not require comparable editing burden; it can adopt the same discipline of defined genomic backgrounds, validated edits, genomic provenance, whole-animal phenotyping, and functional validation. The goal is minimal disease-relevant engineering, not maximal editing.

## 3 Cancer status and the reproductive system in the same host

The first uniquely informative experiment is simple: compare a healthy minipig receiving a clinically relevant anticancer exposure with a cancer-bearing minipig receiving the same regimen. If the reproductive outcomes are indistinguishable, cancer status adds little for that tumor–therapy combination. If they differ, the model can resolve whether tumor burden, inflammatory state, metabolism, endocrine signaling, or treatment pharmacology changes gonadal vulnerability. This is the experimental meaning of cancer status as a coordinate rather than a yes/no label.

Treatment class should be treated as a second experimental coordinate rather than collapsed into a generic “anticancer exposure.” The reproductive toxicity of classical cytotoxic regimens is comparatively well characterized, whereas the effects of targeted agents and immune checkpoint inhibitors remain incompletely defined, with clinical evidence still limited and several signals derived primarily from preclinical or small observational studies (2, 32). This uncertainty is precisely where an immunocompetent cancer-bearing minipig may add value. Targeted therapies can produce pathway-specific effects on gonadal, vascular, endocrine, or stromal compartments, while immune therapies may alter reproductive function indirectly through immune activation, endocrine immune-related adverse events, or changes in tumor-host signaling. Oncopig studies have already demonstrated measurable antitumor immune responses and compatibility with clinically relevant interventional and combination-immunotherapy concepts, supporting its use to interrogate treatment-host interactions rather than drug exposure alone (21). A paired design could compare tumor control, pharmacokinetics/pharmacodynamics, endocrine state, ovarian or testicular injury, and later reproductive competence in healthy versus cancer-bearing animals receiving the same agent.

This design also gives the platform a direct role in individualized risk prediction. Rather than classifying a regimen as simply gonadotoxic or non-gonadotoxic, serial measurements could determine how baseline reproductive reserve, pubertal state, tumor burden, systemic inflammation, endocrine status, and exposure metrics interact to shape outcome. Candidate biomarkers would then be judged prospectively by whether measurements obtained before or early during therapy predict later ovarian, testicular, or gamete function. The goal is not to create another omics catalogue, but to identify clinically interpretable predictors that can improve counselling about whether preservation is urgent, whether tissue-based approaches are justified, and whether a patient may retain a meaningful post-treatment window for additional fertility preservation (1, 2, 32, 33).

Two disease modules follow from this principle. Distant-cancer minipigs would test whether systemic tumor-host biology modifies ovarian or testicular injury, preservation response, and recovery. Reproductive-organ models address the harder problem of cancer and fertility occupying the same anatomical system. High-grade serous ovarian carcinoma and testicular germ-cell tumors have distinct genomic and developmental architectures (34, 35); therefore, the model must reproduce the genotype, cell of origin, and pathology—not merely the organ.

The canonical Oncopig KRAS^G12D^/TP53^R167H^ cassette is not, by itself, a genotype-faithful model of either high-grade serous ovarian carcinoma (HGSOC) or testicular germ-cell tumor (TGCT). This limitation concerns disease-specific genomic fidelity rather than the value of the Oncopig architecture itself. The Oncopig supplies the established large-animal framework—an immunocompetent host, controllable cancer initiation, clinically scaled procedures, longitudinal sampling, and tumor–host interrogation—on which reproductive-organ cancer modules can be built. For HGSOC, a next-generation porcine module would require a fallopian-tube secretory epithelial cell-of-origin strategy with TP53 disruption and a disease-relevant genomic route, such as homologous-recombination deficiency involving BRCA1/2 or a homologous-recombination-proficient copy-number-driven state such as CCNE1 amplification; MYC amplification is recurrent but not an obligatory lesion (34, 36). For TGCT, model construction would require a developmental design rooted in fetal/primordial germ-cell or gonocyte biology, followed by validation of characteristic 12p gain and, where subtype-relevant, KIT/RAS-pathway alterations (35). These reproductive-organ modules therefore require new engineered alleles or chromosomal architectures, lineage-appropriate targeting, and pathology-led validation; changing only the anatomical site of Cre induction would be insufficient. They should be viewed as disease-specific, next-generation extensions of the Oncopig framework rather than replacements for it.

Genome engineering alone does not establish disease fidelity. Comparative experimental pathology should act as a validation layer integrating histomorphology, immunophenotype, molecular pathology, tumor microenvironment, and cross-species concordance. The Oncopig experience illustrates why: direct AdCre induction can yield nonspecific histology, whereas autologous implantation of molecularly characterized, CRISPR-edited porcine cancer cells offers tighter control of phenotype (21, 37). Future ovarian/fallopian and testicular models should be named by demonstrated concordance with the intended human disease, not simply by anatomical site.

This genomic and pathological discipline is clinically relevant. In ovarian and fallopian-tube cancers, experimental models could test whether tissue selected for fertility preservation is free of malignant contamination, whether neoadjuvant therapy changes oncological safety and follicular competence in opposite directions, and whether fertility-sparing surgery preserves function without compromising tumor control. Comparative pathology can distinguish tumor infiltration, treatment-induced injury, background lesions, and recovery phenotypes in preserved ovarian or testicular tissue, linking molecular readouts to tissue-level biological meaning. The risk of reintroducing malignant cells remains a central concern in ovarian-tissue transplantation, and strategies for detection, purging, follicle isolation, and alternative maturation remain under development (38–42). In boys and adolescents unable to bank sperm, testicular-tissue cryopreservation is increasingly offered but remains experimental with respect to restoration of fertility, creating a clear need for models that link oncological safety to long-term spermatogenic function (7).

## 4 A programmable minipig oncofertility platform

We propose a MiniPig Oncofertility Platform organized around five states: reproductive baseline, cancer-bearing state, therapy exposure, fertility preservation, and reproductive recovery (**Figure 1**). Its defining feature is continuity: the same host and the same genomic background are followed across the trajectory. Serial blood, tumor, ovarian or testicular samples, semen, follicular material, imaging, endocrine phenotypes, pharmacokinetics, and cryopreserved tissues can be linked rather than inferred across unrelated experiments. Where an engineered line is generated by SCNT, the definitive longitudinal reproductive trajectory should be centered on conventionally bred, genotype-defined offspring rather than on the founder clone.

**Figure 1:**
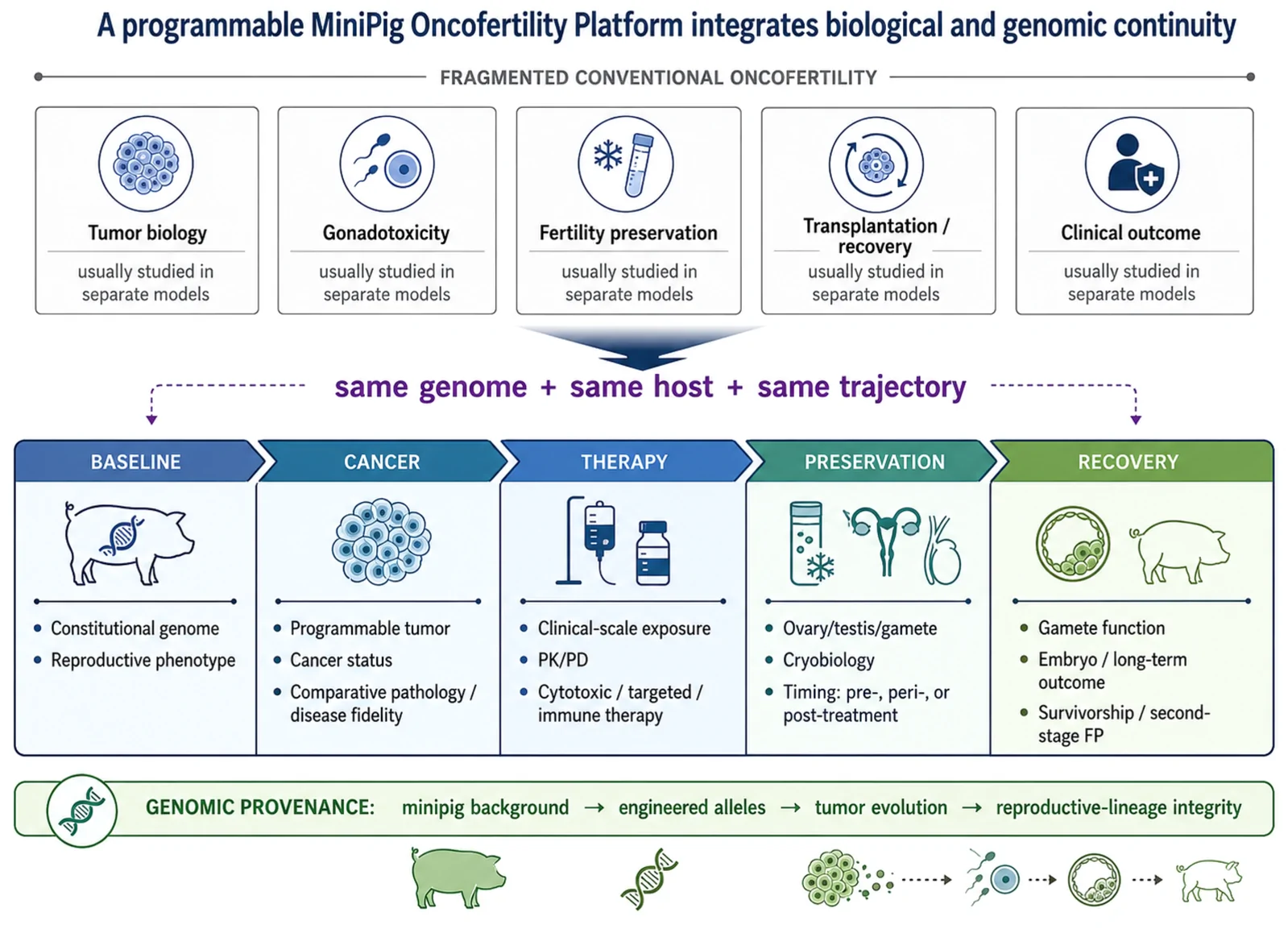
A programmable MiniPig Oncofertility Platform integrates biological and genomic continuity. Conventional experimental oncofertility often investigates tumor biology, treatment-associated gonadotoxicity, fertility preservation, transplantation, and reproductive recovery in separate models. The proposed MiniPig Oncofertility Platform integrates these domains within the same genetically defined host across five longitudinal states: reproductive baseline, cancer-bearing state, therapy exposure, fertility preservation, and recovery/survivorship. The Oncopig framework provides the programmable oncologic backbone and disease context, while comparative experimental pathology serves as a fidelity checkpoint for tumor histology, molecular phenotype, and correspondence with the intended human disease. Clinically relevant cytotoxic, targeted, or immune-based therapies can be evaluated together with pharmacokinetic/pharmacodynamic and reproductive endpoints. Fertility-preservation timing can be interrogated before, during, or after treatment, with outcomes extending from ovarian or testicular integrity and gamete competence to embryo development and long-term reproductive recovery. For engineered lines generated by SCNT, definitive reproductive phenotyping should be performed in germline-derived offspring to minimize founder-generation confounding. Throughout the trajectory, genomic provenance links the constitutional minipig background, engineered alleles, tumor evolution, and reproductive-lineage integrity. The central concept is same genome, same host, same biological trajectory, enabling cancer control and reproductive outcome to be studied as interconnected rather than isolated experimental processes.

Timing of fertility preservation should be encoded within this trajectory as an explicit experimental coordinate, not treated only as a logistical variable. The same platform could distinguish material collected before therapy, during treatment or between treatment cycles when clinically relevant, immediately after treatment, and during survivorship. Each time point represents a different biological state: pretreatment tissue may carry cancer-associated host conditioning; treatment-exposed tissue may contain acute DNA damage, inflammatory or vascular perturbation, and altered follicular or spermatogenic dynamics; later tissue may show partial recovery, persistent depletion, or clonal selection. This design directly addresses one of the unresolved questions in current oncofertility whether there is a meaningful role for fertility preservation after chemotherapy and allows timing to be tested against functional rather than purely morphological outcomes (1, 2, 33).

The most informative experiment would use within-animal or tightly matched longitudinal comparisons whenever feasible. Reproductive tissue or gametes preserved before treatment could be compared with material obtained after a defined regimen from the same genomic background, while serial endocrine measurements, semen parameters, imaging, circulating biomarkers, and pharmacokinetic data establish the intervening trajectory. For ovarian studies, post-treatment material could be evaluated for follicular density, stromal and vascular integrity, oocyte competence, and minimal residual disease where relevant; for testicular studies, spermatogonial survival, niche integrity, spermatogenic recovery, and sperm competence could be followed. A post-treatment sample that appears histologically preserved but fails to support competent gametes would demonstrate why timing cannot be inferred from cell survival alone. Conversely, recovery of function could identify a clinically relevant window for a second-stage preservation strategy in selected survivors (33).

The genomic standard should be genomic provenance rather than simply genotyping. Every experimental animal should have a record linking the reference build and miniature-swine lineage, generation history (SCNT founder versus germline-derived F1/F2), phased germline variation when feasible, exact engineered alleles, on-target architecture, relevant off-target or structural events, serial tumor evolution, and the genomic status of reproductive material selected for preservation. The constitutional genome becomes the anchor against which designed drivers and acquired tumor evolution are interpreted. This is particularly important in oncofertility because an edit acceptable in a somatic cancer model may be unacceptable if unintentionally present in a germline lineage intended for reproduction.

Genomic provenance also creates a bidirectional comparative-oncology workflow. Human tumor genomics can nominate a driver architecture; the minipig genome can be interrogated for orthology, regulatory context, and structural compatibility; the model can then be engineered, pathologically validated, and phenotyped; and the resulting tumor and reproductive signatures can be compared back with human datasets. PigGTEx and chromosome-complete assemblies support tissue-level interpretation beyond simple gene-name matching (10–12). This framework favors minimal, interpretable engineering embedded in maximal genomic knowledge rather than maximal editing for its own sake.

The distinctive value of the minipig is integration rather than superiority in any single experimental dimension. Rodent models remain indispensable for rapid mechanistic discovery and genetic dissection, human tissues for direct species relevance, and clinical cohorts for patient-centered outcomes. The minipig adds a complementary scale for testing mechanisms within clinically relevant anatomy, treatment delivery, longitudinal physiology, reproductive intervention, and functional recovery. **Table 1** summarizes clinical questions for which this integration could be particularly informative.

**Table 1:** Clinical questions enabled or strengthened by a genome-defined MiniPig Oncofertility Platform.

Clinical problem	Experimental module	What the minipig adds	Key question	Critical endpoint	Cross-species interpretability*
Cancer-associated gonadotoxicity	Oncopig framework: distant-cancer module	Cancer-bearing host + clinically scaled therapy + serial sampling	Does cancer status modify reproductive injury relative to healthy-animal exposure?	Ovarian/testicular function; gamete competence; recovery	Partially comparable: gonadal injury/recovery processes are informative; human-equivalent dose thresholds remain unknown.
Ovarian/fallopian cancer	Oncopig framework: disease-specific ovarian/fallopian module	Oncopig-derived framework + disease-specific genotype + cell-of-origin-defined tumor + comparative pathology + fertility-preservation tissue in the same anatomical system	Can tissue be preserved without retaining malignant contamination or losing follicular competence?	Comparative pathology + MRD/oncological safety + follicular/oocyte function	Unknown until disease fidelity is shown: genotype, cell of origin, and pathology must be human-concordant.
Testicular cancer/prepubertal FP	Oncopig framework: disease-specific TGCT module	Oncopig-derived framework + developmentally appropriate genotype/cell-of-origin + comparative pathology + testicular cryobiology + long-term spermatogenic readouts	Can germline potential be preserved when cancer risk and fertility share the same organ?	Comparative pathology + spermatogenic recovery + sperm competence + genomic safety	Partially comparable: spermatogenic stages/recovery are informative, but kinetics and sperm-production efficiency differ by species.
Fertility-sparing intervention	Oncopig framework: reproductive-organ or distant-cancer module	Clinical-scale surgery/imaging and simultaneous cancer assessment	Can reproductive function be protected without compromising tumor control?	Tumor response + endocrine/gamete recovery	Partially comparable: procedural scale is informative; oncological and fertility outcomes still require human validation.
Developmental safety	Optional embryo endpoint	Defined genetic background linked to gametes/embryos	Does preserved fertility retain developmental competence after cancer and treatment?	Blastocyst competence; lineage state; genomic integrity	Known differences: embryo kinetics, implantation, placentation, and gestation differ; use as within-species functional validation.
Modern therapy reproductive safety	Oncopig framework: distant-cancer immunocompetent module	Cancer-bearing immune context + clinically scaled exposure + serial PK/PD and reproductive phenotyping	Do targeted therapies or immune checkpoint inhibitors alter reproductive function through direct or host-mediated mechanisms?	Tumor response + endocrine/gonadal injury + gamete competence	Partially comparable: PK/PD and immune context can be modeled; clinical reproductive-risk thresholds require human calibration.
Timing/post-treatment fertility preservation	Oncopig framework: longitudinal pre- versus post-treatment module	Matched genomic background and sequential reproductive sampling across therapy and survivorship	Does material collected after treatment retain functional and genomic competence comparable with pretreatment material, and is there a recoverable second preservation window?	Functional recovery + gamete/embryo competence + genomic safety	Partially comparable: recovery trajectories are informative; absolute post-treatment timing windows are species-specific.

*Cross-species interpretability is classified conservatively as process-level comparable/partially comparable, unknown until model validation, or a known species difference; these labels refer to the critical endpoint rather than to whole-species equivalence.

## 5 Functional reproductive endpoints and genomic safety

Porcine reproductive biotechnology makes the proposed platform testable. Oocyte *in vitro* maturation and follicle-culture systems provide direct assays of gamete competence: porcine preantral follicles can be grown *in vitro* to yield meiotically competent oocytes capable of fertilization and blastocyst development, although efficiency remains dependent on follicular stage and culture conditions (43). The broader porcine *in vitro* embryo-production literature likewise identifies persistent species-specific limitations, including polyspermy and suboptimal culture conditions, that must be considered when interpreting developmental competence (44).

Ovarian-tissue cryopreservation and xenotransplantation have been studied in prepubertal pigs with follicular, stromal, vascular, and endocrine endpoints (45). Cryopreserved immature porcine testis tissue can retain developmental potential after transplantation, and sperm recovered after cryopreservation and xenografting has produced live piglets (46–48). These endpoints matter because tissue survival, endocrine recovery, gamete production, fertilization, embryo development, and live birth are biologically distinct levels of reproductive restoration. Clinical ovarian-tissue transplantation demonstrates that preserved tissue can restore human fertility, while contemporary guidance emphasizes both the efficacy and oncological-safety constraints of ovarian and testicular tissue preservation (1, 7, 38–40, 42, 49).

A dedicated pediatric/AYA module would be especially well aligned with the translational role of the platform. In prepubertal boys, testicular-tissue cryopreservation is increasingly offered in specialized programs, but restoration of fertility remains investigational and requires animal models capable of linking preservation conditions to long-term spermatogenic function (7). In girls who cannot undergo ovarian stimulation, ovarian-tissue cryopreservation may preserve a large follicular pool but raises age-, diagnosis-, and safety-specific questions that cannot be resolved by short-term viability assays alone. Adolescents and young adults add a different challenge: reproductive potential can evolve during survivorship, and a first preservation decision at diagnosis may not be the last clinically relevant decision (8). The minipig could therefore model not only pediatric tissue preservation but also the transition from prepubertal tissue to pubertal or adult reproductive function under a documented cancer and treatment history.

For survivors, the same framework reframes post-chemotherapy fertility preservation as a biological question rather than a binary recommendation. Recovery can be asynchronous: endocrine markers may normalize while follicular number, spermatogenic output, gamete quality, vascular integrity, or developmental competence remain impaired. Conversely, partial recovery may create a clinically useful interval in which oocyte, sperm, ovarian-tissue, or testicular-tissue preservation becomes feasible after treatment. By sampling the same host across these states, the minipig could test which recovery signals predict usable reproductive function and whether a second intervention adds benefit over observation alone. This is directly relevant to long-term survivor care, where counselling depends not only on what treatment was received, but on how the individual reproductive system recovered over time (8, 33).

The porcine blastocyst can provide a stringent developmental endpoint when embryo generation is scientifically and ethically justified. Single-cell studies resolve lineage allocation and conserved developmental programs in pig embryos, making blastocyst competence molecularly interpretable rather than merely morphological (50, 51). In selected studies, embryos derived from gametes recovered before or after cancer therapy could test whether apparent gonadal recovery extends to developmental competence. This endpoint should remain optional and question-driven, but it provides a functional bridge between tissue preservation and reproductive outcome.

For reproductive-organ cancers, genomic safety becomes inseparable from reproductive efficacy. A preserved ovarian fragment, spermatogonial population, gamete, or embryo should be evaluated not only for viability but also for unwanted engineered alleles, clonal abnormalities, tumor-associated changes, and where relevant minimal residual disease. Genomic provenance therefore connects model construction to the clinical question that ultimately matters: can reproductive function be preserved or restored without compromising oncological safety?

## 6 What would validate or refute the platform?

A transformative-platform claim must be falsifiable. Cancer-bearing and healthy minipigs exposed to the same treatment should first be compared to determine whether cancer status changes reproductive injury. Future ovarian/fallopian or testicular models must then reproduce the intended human histology, cell of origin, molecular state, and tumor microenvironment; anatomical location alone is insufficient. Fertility-preservation strategies should be evaluated simultaneously for reproductive benefit and tumor control. Current Oncopig model-development constraints—including nonspecific histology after direct AdCre induction, spontaneous tumor regression, incompletely established metastatic behavior, and limited porcine-specific reagents—define the validation agenda for next-generation modules and should be treated as explicit validation criteria rather than overlooked limitations (21). Comparative pathology and molecular profiling should therefore accompany model development, especially before survivorship-oriented reproductive claims are made. If simpler biomarkers or models fully predict recovery, additional large-animal complexity is unnecessary.

The platform should be used selectively and in complement with other experimental systems. Human tissues and organoids provide direct species relevance, while rodent models remain essential for rapid mechanistic and genetic studies. The Oncopig addresses a different experimental space: questions that require cancer to coexist with intact immune and endocrine physiology, clinically scaled intervention, longitudinal pharmacokinetics/pharmacodynamics, reproductive procedures, and functional recovery within the same host. Its distinctive value is therefore not generic large-animal complexity, but the integration of these domains within a genomically defined cancer-bearing organism.

A first-generation validation matrix should target the questions for which current evidence is least secure. One arm should use a conventional cytotoxic regimen as a benchmark; a second should evaluate a targeted or immune-based therapy with uncertain reproductive safety; and a third should compare fertility-preservation timing before versus after treatment. Each experiment should pair oncological and reproductive endpoints so that preservation is not judged successful at the cost of tumor control. Recovery should be defined hierarchically—endocrine function, follicular or spermatogenic recovery, gamete competence, embryo competence when justified, and long-term reproductive outcome. Serial biomarkers should be tested prospectively for their ability to forecast later reproductive recovery rather than merely correlate with contemporaneous injury (32, 33).

The field is technically positioned to test this proposition. High-resolution pig genomics provides the coordinate system; CRISPR and SCNT provide causal control; xenotransplantation demonstrates systems-level genome engineering; the Oncopig provides the oncologic backbone for programmable cancer *in vivo*; comparative experimental pathology provides a fidelity checkpoint; and porcine reproductive biotechnology supplies endpoints extending from gonadal tissue and gametes to embryos and offspring (21, 26–29, 37). Together, these capabilities support an Oncopig-centered translational sequence: engineer the disease-relevant genome, establish the cancer state, validate the pathology, and follow reproductive outcome. The platform couples biological continuity (cancer, therapy, preservation, and recovery) with genomic continuity (constitutional background, engineered state, tumor evolution, and reproductive-lineage integrity). Its strongest endpoint will be cross-level concordance linking a defined cancer and treatment state to reproductive injury and, ultimately, functional recovery. This sequence places pathology between genotype and translation, preventing an engineered tumor from being accepted solely because it arises in the correct organ. In this formulation, the Oncopig is the oncologic backbone that enables cancer, host physiology, therapy, fertility preservation, and reproductive recovery to be interrogated as one longitudinal system.

## Data Availability

The original contributions presented in the study are included in the article/supplementary material. Further inquiries can be directed to the corresponding author.
